# Effect of Low Molecular Weight Heparins (LMWHs) on antiphospholipid Antibodies (aPL) – Mediated Inhibition of Endometrial Angiogenesis

**DOI:** 10.1371/journal.pone.0029660

**Published:** 2012-01-03

**Authors:** Silvia D'Ippolito, Riccardo Marana, Fiorella Di Nicuolo, Roberta Castellani, Manuela Veglia, John Stinson, Giovanni Scambia, Nicoletta Di Simone

**Affiliations:** 1 Department of Obstetrics and Gynecology, Università Cattolica del Sacro Cuore, Rome, Italy; 2 Istituto Scientifico Internazionale Paolo VI, Università Cattolica del Sacro Cuore, Rome, Italy; 3 LEO Pharma, Ballerup, Denmark; National Cancer Center, Japan

## Abstract

Antiphospholipid syndrome (APS) is an autoimmune disorder characterized by vascular thrombosis and/or pregnancy morbidity in the presence of circulating antiphospholipid antibodies (aPL). Different pathogenic mechanisms for aPL-mediated pregnancy failure have been proposed. In particular a direct effect of aPL on both maternal and fetal side of the placental tissue has been reported, since their reactivity with β2-glycoprotein I (β2GPI) makes them adhere to trophoblast and human endometrial endothelial cell (HEEC) membranes. β2GPI can be recognized by aPL that, once bound, interfere with both trophoblast functions and with the HEEC differentiation.

APS patients can be successfully treated with Low Molecular Weight Heparin (LMWH). Recent reports suggest that LMWH acts through mechanisms alternative to its well known anticoagulant effect, because of its ability to bind β2GPI. In our previous studies, we showed that LMWH is able to reduce the aPL binding to trophoblasts and restore cell invasiveness and differentiation. So far, however, no study has described its effects on endometrial angiogenesis.

The aim of our research was to evaluate whether two LMWHs, tinzaparin and enoxaparin, have an effect on the aPL-inhibited endometrial angiogenesis. This prompted us to investigate: (i) *in vitro* HEEC angiogenesis through a Matrigel assay; (ii) VEGF secretion by ELISA; (iii) matrix metalloproteinase-2 (MMP-2) activity by gelatin zymography; (iv) Nuclear Factor-κB (NF-κB) DNA binding activity by colorimetric assay; (v) STAT-3 activation by a sandwich-ELISA kit. Furthermore, using an *in vivo* murine model we investigated the LMWHs effects on angiogenesis.

We demonstrated that the addition of LMWHs prevents aPL-inhibited HEEC angiogenesis, both *in vitro* and *in vivo*, and is able to restore the aPL inhibited NF-κB and/or STAT-3 activity, the VEGF secretion and the MMPs activity.

The demonstration of a beneficial role for LMWHs on the aPL-inhibited HEEC angiogenesis might provide additional mechanisms whereby this treatment protects early pregnancy in APS.

## Introduction

“Obstetric” Antiphospholipid Syndrome (APS) refers to pregnancy morbidity occurring in patients with persistent antiphospholipid antibodies (aPL) [1]. Obstetric criteria used to define APS are: fetal loss after 10 weeks' gestation, three or more early miscarriages (unexplained consecutive embryonic losses before the 10th week of gestation), preeclampsia or features of placental insufficiency, associated with the premature birth of a morphologically normal neonate before the 34th week of gestation [2], [3], [4], [5]. Thrombosis in the placental vasculature was initially thought to be the main cause of adverse pregnancy outcome in the syndrome [4]. However, the heterogeneity of histological lesions in APS placentas suggested that intraplacental thrombosis was unlikely to be responsible for all of the aPL-associated poor obstetric outcomes [6], [7]. Whereas, a direct effect of aPL on trophoblast cells has been demonstrated through *in vitro* and animal studies [6], [7], [8], [9], [10], [11]. Indeed (i) the demonstration of the expression of β2-glycoprotein I (β2GPI) on trophoblast cell membranes; (ii) the aPL ability to bind trophoblast monolayers *in vitro* and to negatively affect trophoblast cell functions; (iii) the raised complement activation and the increased secretion of Tumor Necrosis Factor (TNF)-α and chemokines observed in *in vivo* murine models of APS, all provided important insights into the pathophysiology of pregnancy morbidity in APS [8], [9], [10], [11].

Our recent studies also demonstrated aPL's ability to affect the maternal side of the placenta by directly binding human endometrial endothelial cells (HEEC) [12]. As a consequence, aPL induced a significant decrease in both number and total length of capillary structures formed by HEEC in an *in vitro* Matrigel assay [12]. We confirmed this inhibitory effect *in vivo* through a murine model [12]. These observations indicate that aPL act through multiple pathogenic mechanisms, such as decreased trophoblast invasion and impaired HEEC differentiation, which altogether might interfere with physiological placentation and explain APS pregnancy complications.

Low molecular weight heparins (LMWHs) are widely used in the management of APS patients [13], [14]. Consistent with the initial aetiological thrombotic theory, this therapy focused on preventing thrombosis. However, the presence of alternative mechanisms of placental damage in APS and the success of heparin treatment on pregnancy outcome stimulated interest on the drug's mechanism of action. Accordingly, the protective effects of heparin have been related to its ability to prevent the binding of aPL to trophoblast cell membranes and to reduce the local aPL-induced complement activation at various points in the classical, alternative and terminal pathways [6], [7], [15], [16], [17].

Given these observations, the objective of our study was to evaluate whether two different LMWHs, tinzaparin and enoxaparin, have an effect on the aPL-inhibited endometrial angiogenesis both *in vitro* and *in vivo*. An additional aim was to investigate the regulation of intracellular HEEC-signalling mechanisms in presence of aPL with or without increasing doses of these LMWHs. Any demonstration of the beneficial role of LMWHs on the aPL-inhibited HEEC angiogenesis might provide additional mechanisms by which this treatment protects early pregnancy in APS patients.

## Materials and Methods

### Antibody preparation

Polyclonal aPL were isolated from patients with APS, diagnosed according to the revised Sapporo criteria [5]. All patients provided informed consent for participating in the study. The study was approved by the human investigation committee of the Università Cattolica del Sacro Cuore (Rome, Italy).

IgGs were purified by affinity chromatography using protein G-Sepharose chromatography columns (Amersham Biosciences, GE Healthcare, CH) [11]. The final protein IgG concentration was evaluated by nephelometry and the specific reactivity with CL and β2GPI-coated plates was confirmed as previously described [18]. Sterile-filtered IgG fractions were determined to be free of endotoxin contamination by the limulus amoebocyte lysate assay (E-Toxate, Sigma Chemical Co., St. Louis, MO; sample sensitivity <0.03 IU/µl).

### Tissue collection

Endometrial tissues were obtained from fertile women (in the mid secretory phase) undergoing hysterectomy for fibroid uterus or biopsy (n = seven biopsies per procedure) for benign diseases (written informed consent was obtained from each patient before the surgery). The mean age of the patients was 37.4 years, range 30–42 years. The day of the menstrual cycle was determined by the patients' menstrual history and was verified through the histological examination of the endometrium according to Noyes criteria [19]. Estradiol and progesterone were determined in the serum to confirm the mid secretory phase. The tissues were placed in Hank's balanced salt solution (HBSS) and carried to the laboratory for HEEC isolation and culture. Each experimental setup was repeated on at least five occasions using cells obtained from different patients.

### HEEC isolation and purification

The endometrium was minced and incubated in M199/penicillin/streptomycin containing 0,2% collagenase type II at 37°C for 2 hours (hrs). At the end, all remaining tissue was dissolved by powerful resuspension, resulting in a homogenous solution. After centrifugation (1200 rpm for 5 min at room-temperature), the pellet obtained was resuspended in culture medium and transferred into a fibronectin-coated culture dish. After 2–4 hrs, the non-adhered cells were removed and the adherent cells were cultured in human endometrial microvascular endothelial cells (hEMVEC) culture medium.

The primary heterogeneous cell population was grown until near confluence before selection of the endothelial cells using anti-human CD31 and CD105 coated microbead (Miltenyi Biotec S.r.l, Italy). In brief, after detachment using trypsin and centrifugation, the cells were immunolabelled with CD31 and then CD105 MicroBeads (20 µl per 1×10^7^ cells) before being loaded onto a column placed in the magnetic field of a MACS Separator. The magnetically labeled CD31^+^/CD105^+^ cells were retained on the column, whereas, the unlabelled cells passed through. After removing the column from the magnetic field, the positive CD31^+^/CD105^+^ magnetically retained cells were eluted and taken directly into culture or analyzed for purity by flow cytometry.

### Flow cytometry

**Figure 1:**
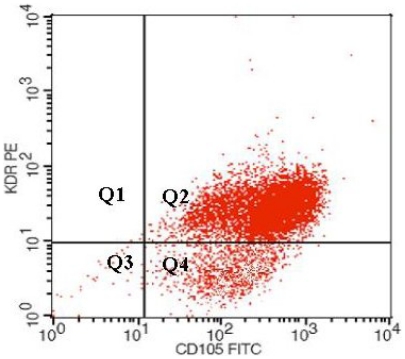
HEEC characterization. Endometrial samples were subjected to CD31 and CD105 cell immunomagnetic isolation by incubation with anti-CD31 and anti-CD105 conjugated-microbeads, and subsequent loading on a MidiMACS device. Cell viability after immunoselection exceeded 98% in all cases, as judged by propidium iodide staining by flow cytometry. Cells recovered were analyzed for the presence of endothelial markers (Vascular Endothelial Growth Factor Receptor-2 or KDR and CD 105) by flow cytometry analysis (KDR-PE/CD105-FITC = 97%).

Characterization of the isolated HEEC was performed by flow cytometry. Briefly, aliquots of HEEC were incubated for 15 min at room-temperature with specific endothelial markers: FITC- or APC- conjugates monoclonal antibodies (MoAbs) VE-cadherin and/or KDR (R&D System, Abingdon, UK). Appropriate fluorochrome-conjugated isotype-matched irrelevant MoAbs were used as control for background staining. Cells were run through a flow cytometer (FACSCanto, Becton Dickinson, Mountain View, CA, USA). A minimum of 10,000 events were collected and acquired in list mode with the accompanying software (FACS Diva software, Becton Dickinson; **Figure 1**). Human Umbilical Vein Endothelial Cells (HUVEC, American Type Culture Collection, ATCC, USA) were used as positive control (data not shown).

### *In vitro* Angiogenesis Assay

Endothelial cell differentiation into capillary-like tube structures was monitored by BD Biocoat angiogenesis system (BD Biosciences). HEEC were seeded on Matrigel-coated plates (2×10^4^ cell/wells) in endothelial cell differentiation culture medium (EBM-2) MV Single Quots (Lonza, Milan, Italy) containing aPL (50 µg/ml) in combination with either tinzaparin (0.1–10 IU/ml, innohep®, LEO Pharma A/S, Ballerup, Denmark) or enoxaparin (0.1–10 IU/ml, Clexane, Sanofi SpA, Milan, Italy) and incubated for 8–12 hrs at 37°C, in a 5% CO_2_ atmosphere. Suramin 40 µM (Calbiochem, San Diego, CA, USA) was used as a negative control.

Following incubation, the plates were washed twice with HBSS and the tube formation was observed using an inverted phase optical microscope (Olympus, IX50, Milan, Italy). Images were acquired with a digital camera (Nikon, Tokyo, Japan) and quantified by Photoshop software (San Jose, CA, USA) measuring the number and the total length of the tubules within each well.

### Measurement of Nuclear Factor-κB (NF-κB) DNA binding activity

DNA binding activity of NF-κB was measured with a sensitive multiwell colorimetric assay (Transcription Factor Assay kit; Millipore, Temecula, USA). In short, HEEC cultured in endothelial cell differentiation medium (EBM-2) MV Single Quots were scraped and centrifuged for 10 minutes at 1,500 rpm. The pellet was resuspended in 100 µl of lysis buffer and the lysate was centrifuged for 20 minutes at 15,000 rpm. The supernatant represented the total protein extract and the remaining pellet contained the nuclear portion of cell lysate. The nuclear pellet was resuspended in ice-cold nuclear extraction buffer for 30–60 minutes at 4°C and then centrifuged at 16,000 rpm for 5 minutes. The nuclear extracts in the supernatant (5 µg/5 µl) from each sample were incubated in 96-well plates coated as follows: with NF-κB consensus double-stranded oligonucleotide sequence (5′-AGTTGAGGGGACTTTCCCAGGC-3′) for 1 hr, then with primary NF-κB antibody (1∶500) for 1 hr, subsequently with peroxidase-conjugated secondary antibody (1∶500) for 1 hr, and finally with peroxidase-conjugated secondary antibody (1∶1000) for 1 hr at room-temperature. After colorimetric reaction, optical density was read at 450 nm. For competition assays, cell extracts were incubated with 22-bp double-stranded DNA, either wild-type or mutated (5′- AGTTGAGCTCACTTTCCCAGGC-3′; underline denotes the substitution). Competition experiments performed with a 100-fold excess of unlabeled KB oligonucleotide demonstrated the specificity of the DNA binding activity.

### Measurement of STAT-3 phosphorylation

STAT-3 phosphorylation in presence of aPL with, or without, LMWHs was examined using a sandwich-ELISA kit (PathScan® Phospho-Stat3 (Tyr705) Sandwich ELISA; Cell Signaling Technology Inc., Danvers, MA, USA) according to the instructions provided by the manufacturer. Briefly, cells were lysed using ice-cold lysis buffer and the lysates were further sonicated on ice. Then, 100 µl of the respective lysates were added to a microplate well and incubated at 37°C for 2 hrs. After incubation both non phospho- and phospho- -STAT3 proteins were captured by the coated antibody. Following extensive washing, a phospho-STAT3 mouse monoclonal antibody was added to detect the captured phospho-STAT3 protein. HRP-linked anti-mouse antibody was then used to recognize the bound detection antibody. HRP substrate, TMB, was added to develop colour. The magnitude of optical density for this developed colour was proportional to the quantity of phospho-STAT3 protein (absorbance of each well was measured at λ = 450 nm).

### Vascular Endothelial Growth Factor (VEGF) secretion

VEGF secretion was determined by a human VEGF colorimetric ELISA kit (Pierce Endogen, Rockford, USA) according to the manufacturer's instructions. Briefly, HEEC were plated in 24-well plates at 50,000 cells/well in endothelial cell culture medium with 5% FBS containing aPL (50 µg/ml) with or without LMWHs (tinzaparin or enoxaparin, 0.1–1.0 IU/ml) and incubated for 24 hrs at 37°C and 5% CO_2_ atmosphere. Culture medium or standard (50 µl) was added to each well, previously coated with human monoclonal anti-VEGF antibody. After 2 hrs of incubation, wells were washed and incubated with an enzyme-linked polyclonal anti-VEGF antibody. 3,3′,5,5′-Tetramethyl benzidine substrate solution (TMB) was added to each well and the colour developed in proportion to the amount of the VEGF bound in the initial step. The plate was read on a Titertek Multiscan plus Mk II plate reader (ICN Flow Laboratories, Irvine, CA, USA) by measuring the absorbance at a wavelength of 450 nm minus 550 nm.

### Matrix Metalloprotease (MMP)-2 activity

MMP-2 levels in the supernatant of HEEC cultures were measured by gelatine zymography. Samples were electrophoresed on SDS-polyacrylamide gel containing 0.3% gelatin. Following electrophoresis, gels were washed 3 times in 2.5% Triton X-100 for 10 min at room-temperature, to remove SDS. After overnight incubation at 37°C in 50 mM Tris-HCl (pH 7.4, containing 5 mM CaCl_2_, 0.15 M NaCl and 0.02% NaN_3_), gels were stained with 0.5% Coomassie Brilliant Blue for 30 minutes and then destained in 20% methanol and 10% acetic acid. Gelatinolytic activities were observed as clear bands of digested gelatine on a blue background. Images were acquired with a digital camera (Nikon, Tokyo, Japan) and bands were analyzed on the Image Analysis System Gel Doc 200 System (Bio-Rad Laboratoires) by using Quantity One Quantitation Software (Bio-Rad Laboratoires).

### Angiogenesis by Direct *In Vivo* Assay

Five-week-old CD1 female nude mice obtained from an outbreed background were purchased from Charles River Laboratory. The housing and handling of these mice were in accordance with institutional guidelines and compliant with national (Ministry of Health, Rome, Italy) and international regulation (European Community and National Institutes of Health, Bethesda, MD). All experimental procedures were approved by the local ethical committee on preclinical studies (Commissione per la Valutazione Etica di Sperimentazioni Animali e di Correttezza della Gestione dell'“animal care”. Approval ID: HH 13). The mice were allowed to acclimatise to their new environment for one week. They were housed in disinfected polycarbonate mouse cages, maintained in a cabinet with laminar flow at 28°C under controlled artificial lighting (12 hrs light/12 hrs dark), and given ad libitum access to rodent chow and water during the study. We performed the experiments on groups of 10 animals. For direct angiogenesis assay, the direct *in vivo* assay (DIVA) kit was used (Trevigen, Inc., Gaithersburg, MD, USA). Briefly, angioreactors were filled with Matrigel with, or without, the angiogenic factor (Fibroblast Growth Factor-2, FGF-2), and polyclonal aPL (50 µg/ml) with, or without, different concentration of LMWHs. They were incubated at 37°C for 1 hr to allow gel formation, before subcutaneous implantation into the dorsal flank of the mice. In each mouse, two angioreactors were implanted: the positive control (angioreactor coated with FGF-2) in the left flank and the angioreactor with FGF-2 and aPL (50 µg/ml) with or without tinzaparin in the right flank. At the end of the experiment, the angioreactors were collected and the new vessel formation was determined by FITC-lectin staining. After staining, the vessels were washed and the fluorescence was measured in 96 multiwell plates using a spectrofluorimeter reader (Twinkle LB 970, Berthold Technologies. excitation, 485 nm; emission, 510 nm). The mean relative fluorescence ± SE was determined for five replicate assays.

### Statistical Analyses

The results are expressed as the means ± standard error (SE). One way analysis of variance (ANOVA) was used to determine significant differences among groups. When appropriate, a post-hoc test (Bonferroni test) was used to determine the significance of difference between pairs of means. Statistical significance was accepted at P<0.05.

## Results

### HEEC characterization

Five different endometrial samples were incubated with anti-CD31 and anti-CD105 conjugated-microbeads, and subsequent loaded on MidiMACS device. Cell viability after immunoselection exceeded 98% in all cases, as judged by propidium iodide staining by flow cytometry.

Cells recovered were analyzed for the expression of endothelial markers (VEGF Receptor-2 or KDR and CD105 by flow cytometry analysis (KDR-PE/CD105-FITC = 95.71.%).

### *In vitro* angiogenesis

**Figure 2:**
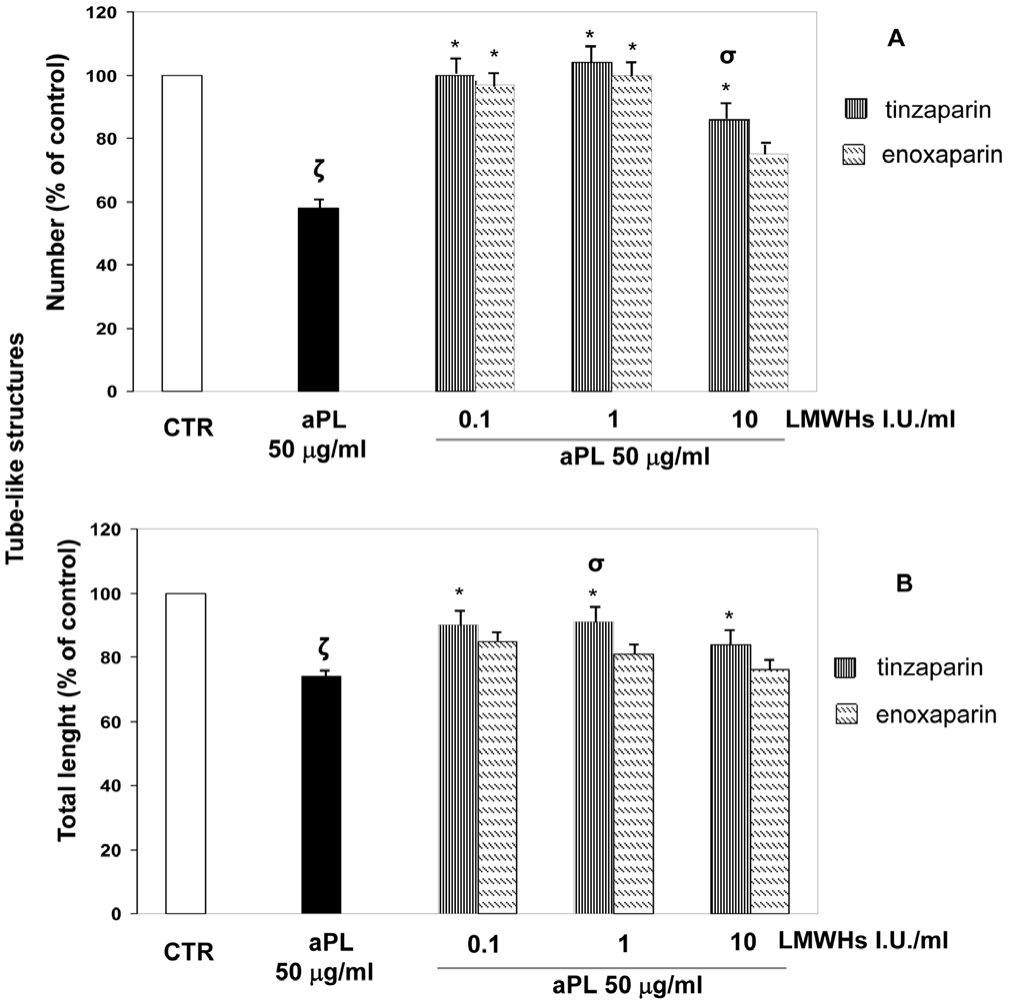
*In vitro* angiogenesis assay. To evaluate the effects of tinzaparin or enoxaparin on aPL-inhibited angiogenesis we used an *in vitro* assay of human endometrial endothelial cells (HEEC) capable of forming tube-like structures in response to the extracellular matrix protein, Matrigel, in endothelial cell culture medium (EBM-2) MV Single Quots. Cells were seeded in matrigel coated plates and examined for tube formation microscopically. The figure shows the quantitative analysis of number (A) and total length (B) of tube-like structures after treatment with aPL (50 µg/ml) with or without tinzaparin or enoxaparin (0.1–10 IU/ml). Results are means ± SE of five experiments and expressed as % of control (CTR = 100). (CTR: untreated cells; **ζ**: P<0.05 compared with CTR; *: P<0.05 compared with aPL; **σ**: P<0.05 enoxaparin vs tinzaparin).

The number (**Figure 2A**) and total length (**Figure 2B**) of the tubules were examined by microscopy. We observed that the addition of tinzaparin and enoxaparin significantly abrogated the aPL-mediated inhibition of tube formation. (**ζ**: P<0.05 compared with CTR; *: P<0.05 compared with aPL). We reported the significant difference between tinzaparin and enoxaparin effect (**σ**: P<0.05 enoxaparin vs tinzaparin).

### Intracellular mechanisms regulating VEGF expression in HEEC. Effects of aPL and LMWHs on NF-κB and STAT-3 activation

**Figure 3:**
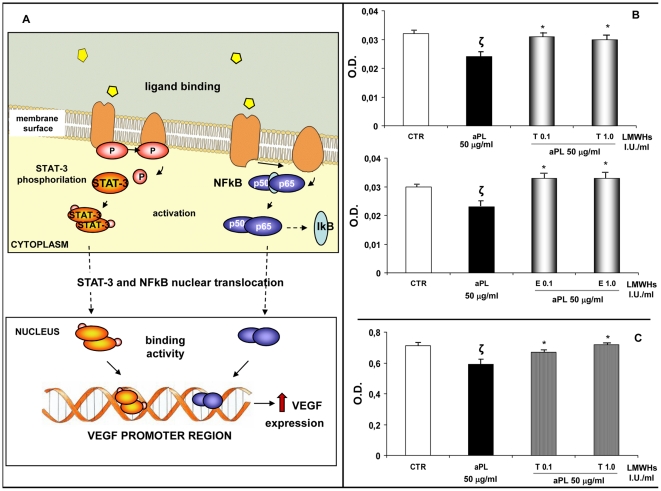
Intracellular mechanisms regulating VEGF expression in HEEC. Effects of aPL and LMWHs on NF-κB and STAT-3 activation. **A.** VEGF is a well-known factor able to promote endothelial cell proliferation and new vessel formation. Several intracellular mechanisms regulate VEGF expression in HEEC including NF-kB and STAT-3. The figure illustrates these two signalling pathways whose activation increases VEGF expression. NF-κB activity is regulated by the interaction with the inhibitory IκB protein. Upon activation, IkB is phosphorylated and degraded, thus allowing NF-κB to translocate into the nucleus and bind to VEGF promoter region, upregulating the expression of this proangiogenic factor by HEEC. Moreover STAT-3 is a member of JAK-STAT signalling pathway. It is a latent transcription factor which is activated by phosphorylation. Activated STAT-3 protein induces its nuclear translocation and favours VEGF expression. **B.** NF-κB activation in the presence of aPL alone or with tinzaparin or enoxaparin after 4 hrs of treatment in differentiation culture medium. aPL reduced NF-κB activation while tinzaparin or enoxaparin were able to restore the NF-κB DNA binding activity. The values are O.D. mean ± SE of three different experiments. CTR: untreated cells; T: tinzaparin; E: enoxaparin; O.D.: Optical Density; **ζ**: P<0.01 compared with CTR; *: P<0.001 compared with aPL-treatment. **C.** Effects of aPL on STAT-3 phosphorylation in the presence of tinzaparin after 4 hrs of treatment in differentiation culture medium. aPL reduced STAT-3 activation while tinzaparin was able to restore the STAT-3 phosphorylation. Values are O.D. means ± SE of three different experiments. CTR: untreated cells; T: tinzaparin; O.D.: Optical Density; ζ: P<0.05 compared with CTR; *: P<0.05 compared with aPL treatment.

**Figure 3A** summarizes NF-κB and STAT-3 signalling pathways, two intracellular mechanisms independently activated during HEEC angiogenesis [20], [21], [22], [23], [24]. The effects of LMWHs on aPL inhibited NFκB binding activity and activation of STAT-3 in presence of aPL with, or without, LMWHs were evaluated. We observed the following: ***(i)*** aPL-mediated inhibition of NF-κB binding activity was stopped by the presence of tinzaparin or enoxaparin (0.1–1.0 IU/ml) (**Figure 3B**; **ζ**: P<0.01 compared with CTR; *: P<0.001 compared with aPL-treatment). *(ii)* aPL (50 µg/ml) inhibited STAT-3 phosphorylation and the addition of tinzaparin (0.1–1.0 IU/ml) to the cultures prevented the aPL-mediated inhibition (**Figure 3C**; **ζ**: P<0.05 compared with CTR; *: P<0.05 compared with aPL treatment), whereas no effect was observed after enoxaparin treatment (data not shown)).

### VEGF and MMP-2 secretion

**Figure 4:**
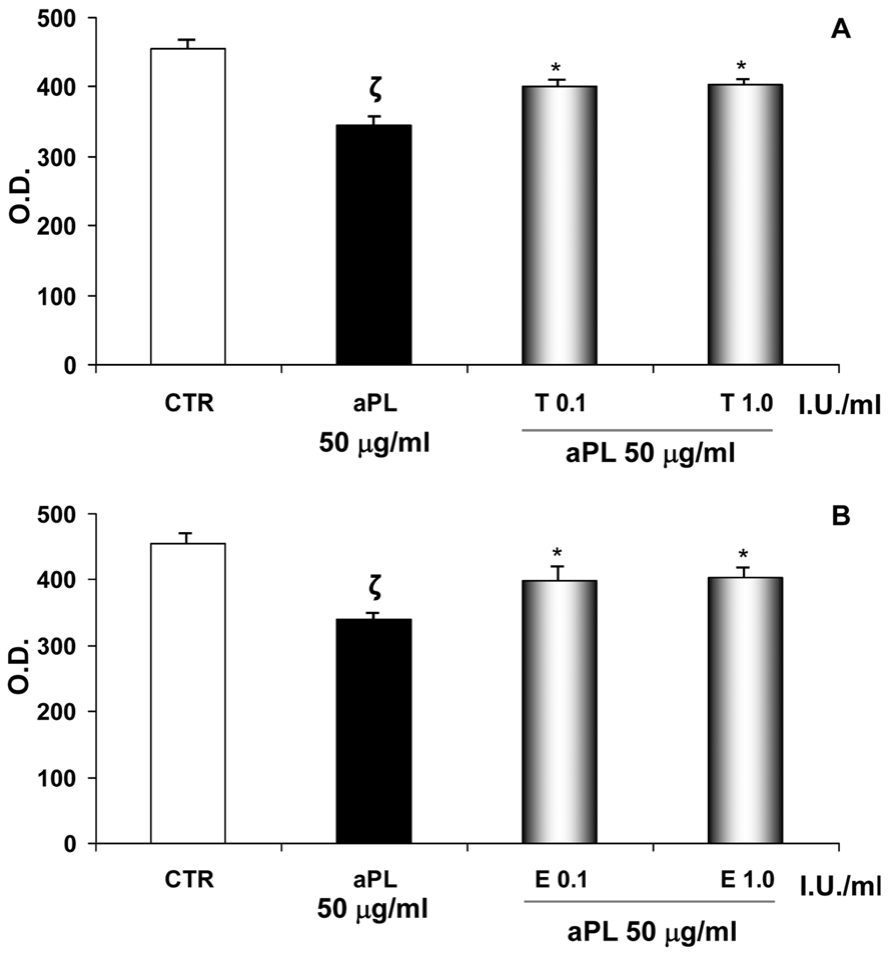
VEGF expression. VEGF secretion in HEEC culture medium was determined by a human VEGF colorimetric ELISA kit. Supernatant samples were collected after 24 hrs of aPL (50 µg/ml) and tinzaparin (A) or enoxaparin (B) (0.1–1.0 IU/ml) treatment for VEGF measurement. Results are mean ± SE of four independent experiments and are expressed O.D. (Optical Density). CTR: untreated cells; T: tinzaparin; E: enoxaparin; O.D.: Optical Density; I.U.: International Units; **ζ**: P<0.05 compared with CTR; *: P<0.01 versus aPL treatment.

**Figure 5:**
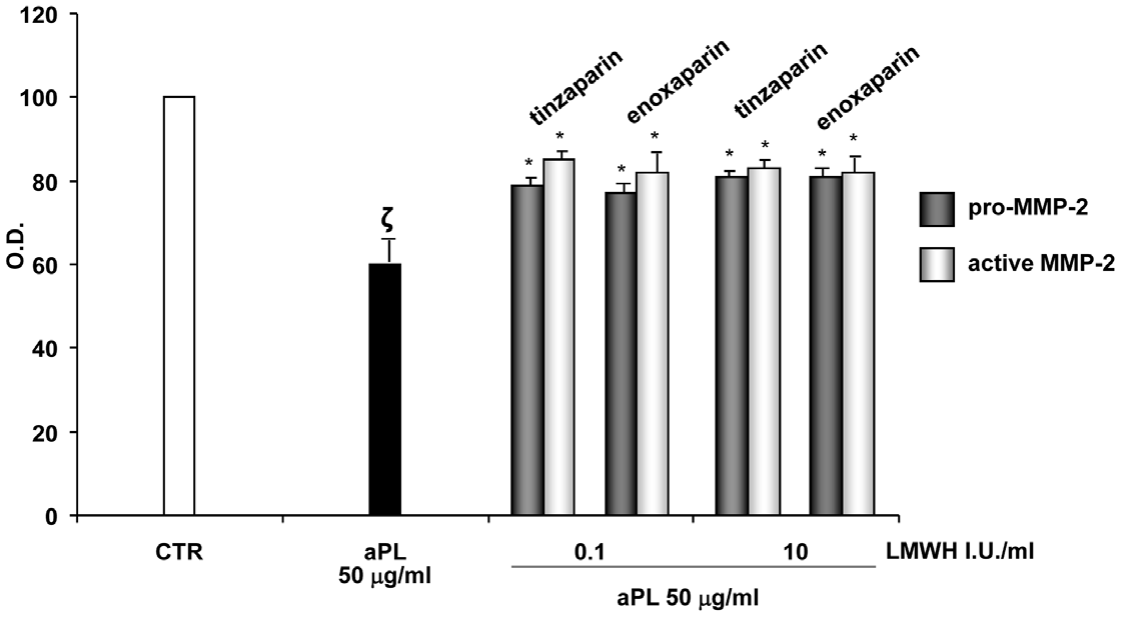
MMP-2 activity evaluation by gelatine zymography. Influence of aPL (50 µg/ml) with or without tinzaparin or enoxaparin treatment on pro-MMP-2 and active MMP-2 gelatinolytic capacity in the supernatant of HEEC. Results are means ± SE from five experiments and expressed as % of control (CTR). CTR: untreated cells; O.D.: Optical Density; I.U.: International Units; ζ: P<0.05 compared with CTR; *: P<0.05 versus aPL treatment.

As shown in **Figure 4**, aPL (50 µg/ml) reduced VEGF production and such inhibition was blocked by the addition of tinzaparin (A) or enoxaparin (B) at a dose of 0.1 and 1.0 IU/ml (**ζ**: P<0.05 compared with CTR; *: P<0.01 versus aPL treatment). Moreover, addition of tinzaparin or enoxaparin HEEC (0.1–1.0 IU/ml) significantly blocked the aPL-inhibited pro- and active-MMP-2 secretion in HEEC culture supernatant, as evaluated by gelatine zymography (**Figure 5**; ζ: P<0.05 compared with CTR; *: P<0.05 versus aPL treatment).

### *In vivo* angiogenesis

**Figure 6:**
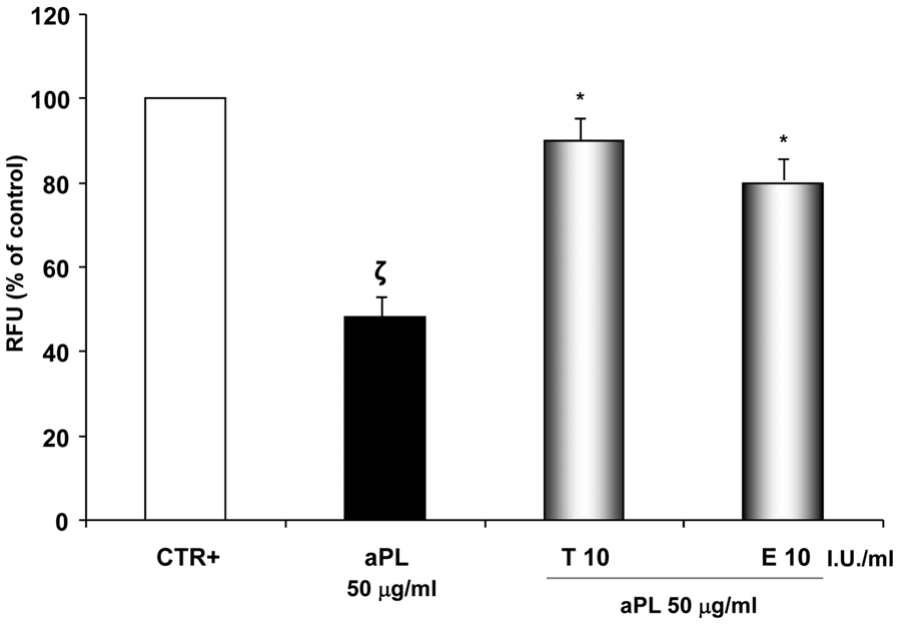
*In vivo* angiogenesis assay. Effects of aPL with or without LMWHs on angiogenesis process *in vivo*. Endothelial cells in the angioreactors were incubated with FITC-lectin, recovered and analyzed for FITC-lectin by fluorescence spectrometry. The analysis demonstrated reduced fluorescence (45%) in angioreactors containing aPL (50 µg/ml) compared with the positive controls. Tinzaparin or enoxaparin were able to completely reverse this angiogenesis inhibition. Results are means ± SE (n = 5 mice per group) from three independent experiments and expressed as RFU (Relative Fluorescent Units), % of control. CTR+: positive control; T: tinzaparin; E: enoxaparin; I.U.: International Units; **ζ**: P<0.05 compared with CTR; *: P<0.05 compared with aPL treatment.

In the presence of tinzaparin or enoxaparin, aPL-inoculated mice revealed a significant increase in new vessel formation compared to the group of mice inoculated with aPL alone (**Figure 6**; **ζ**: P<0.05 compared with CTR; *: P<0.05 compared with aPL treatment). Results are means ± SE (n = 5 mice per group) from three independent experiments. These observations confirmed the LMWHs ability to abrogate the aPL–mediated inhibition of angiogenesis.

## Discussion

In the present study we demonstrated that two different LMWHs, enoxaparin and tinzaparin, are able to prevent the aPL-mediated inhibition of HEEC angiogenesis both *in vitro* and *in vivo*. The LMWHs positive effects involve the up-regulation of signalling pathways and transcriptional factors which are down-regulated in the presence of aPL.

Antiphospholipid antibodies represent a heterogenous group of autoantibodies which do not recognize phospholipids (PL) directly, but via a phospholipid-binding protein: β2GPI. The latter is a highly glycosylated 50-kDa protein consisting of 326 amino acids organized in five domains [25], [26], [27]. Each domain consists of 60 amino acids, with the exception of domain V, which consists of 82 amino acids forming a hydrophobic loop [26], [27]. This specific structure of domain V is responsible for the binding properties of β2GPI to anionic PL [26], [27]. It has been reported that LWMH recognizes a positively charged site within the V domain of β2GPI that is also the same site that interacts with PL [28]. Thus heparin functions as a competitive inhibitor for the PL-binding site and presumably, through this mechanism, ultimately interferes with the binding of aPL to targeted cells [28]. Accordingly, in previous investigations we demonstrated that LMWH reduces the aPL-binding to trophoblast cells and restores *in vitro* placental invasiveness and differentiation [15], [17], [29]. Therefore, we drew the conclusion that heparin, by preventing the binding of β2GPI to negatively charged PL, is able to prevent the deposition of the anti-β2GPI antibodies in tissues and -as a consequence- protect trophoblast PL from the aPL mediated effects during early pregnancy [8], [29].

Subsequent studies, performed on murine models of APS, demonstrated that treatment with heparin prevents complement activation *in vivo* and protects mice from pregnancy complications induced by aPL [16], [30], possibly through its anti-inflammatory action, being able to prevent leukocyte influx/attachment to activated endothelium and to inhibit proinflammatory cytokine secretion [30], [31]. Altogether these findings prove the multiple protective effects of LMWH against aPL-induced placental damage, which are in addition to its well-known anticoagulant properties. However, to date, it was not known whether LMWHs have a possible role on the maternal/endometrial side of human placenta. With this in mind, in our study we evaluated whether two different LMWHs, tinzaparin and enoxaparin, might affect the aPL-inhibitory action on HEEC by modulating some of the most known intracellular pathways that regulate the process of angiogenesis.

During angiogenesis, a coordinated series of steps allows HEEC to invade, migrate and proliferate into the underlying interstitial matrix and form a new capillary structure [20], [21]. A key player in this process is represented by VEGF whose function is to promote the survival, migration and differentiation of endothelial cells, as well as to mediate vascular permeability [32], [33]. Two of the most important intracellular pathways, which finely control VEGF expression and are activated independently during angiogenesis, are NF-κB and STAT-3 [20], [21], [22], [23]. NF-κB is a critical transcriptional factor recognizing multiple regulatory elements in the VEGF promoter region. NF-κB activity is regulated by the interaction with inhibitory IκB protein. Upon activation, IκB is phosphorylated and degraded, thus allowing NF-κB to translocate to the nucleus and bind with VEGF promoter region, upregulating the VEGF expression in HEEC. In turn, VEGF stimulates the expression and activity of MMP-2, a proteolytic enzyme degrading the basement membrane [20], [21], [22], [23], [24], [34]. On the other hand, STAT-3 is a latent transcription factor, member of the JAK-STAT signalling pathway, which upon phosphorylation/activation translocates to the nucleus and favours VEGF expression [24]. Consistent with our previous results, we observed that aPL are able to decrease HEEC angiogenic functions *in vitro* and *in vivo*. Such inhibition is well correlated with an impaired NF-κB activation, VEGF secretion and MMP-2 activity. In addition we showed that LMWH treatment prevented the aPL-mediated inhibition of HEEC angiogenesis both *in vitro* and *in vivo* and we suggest the increased activation of NF-κB and/or STAT-3 as a possible mechanism by which tinzaparin and enoxaparin might restore HEEC angiogenic capacity. The angiogenic effect was observed starting from a dose as low as 0.1 I.U/ml. We can speculate that this action might be due to the peculiarity of our model in which the drug is directly delivered to the cell surface. Growing evidence suggests that failure in the development of a functional placental vasculature, due to an imbalance between placental pro-angiogenic factors (such as VEGF) and anti-angiogenic factors, leads to defective placentation and thus to severe obstetrical consequences such as recurrent pregnancy loss and preeclampsia [33], [35], [36]. Accordingly, we have demonstrated that aPL reduce VEGF secretion by HEEC and that LMWH prevents this inhibitory effect, suggesting a further mechanism by which LMWH protect placental tissue from aPL negative actions.

Several studies have described the inhibitory effect of LMWHs on angiogenesis both *in vitro* and *in vivo*, suggesting that this action is not exclusively related to their anticoagulant function, but perhaps to their interference with the activity of angiogenic growth factors or proteolytic enzymes, to the binding to ECM components, or to the potential effects on pericytes [37], [38], [39]. In preliminary experiments, we confirmed these reports, observing that both tinzaparin and enoxaparin reduced, in a dose-dependent manner, HEEC angiogenesis both *in vitro* and *in vivo* (data not shown). However, the purpose of our study was not to evaluate the role of LMWHs on endometrial angiogenesis, but to examine whether treatment with increasing doses of LMWHs might interfere with the aPL-inhibited HEEC angiogenic behaviour. Antiphospholipid antibodies have been shown to recognize β2GPI expressed on endothelial cells and, once bound, to impair cellular functions [40], [41]. Our data prove that LMWHs are able to antagonise the aPL-mediated effects on HEEC and suggest that such action may be due to the LMWHs' ability to disrupt the interaction of β2GPI with PL on the surface of HEEC.

A noteworthy aspect of our results is that primarily tinzaparin improves aPL-inhibited *in vitro* angiogenesis and STAT-3 activity. It is difficult to explain this difference and caution is necessary in extrapolating our obtained results. Indeed we used doses of tinzaparin and enoxaparin measured in international units, which are established according to their anti-Xa activity. However, this activity is independent of the biological effect examined in our study. Thus, using the same dose for both drugs does not mean that one drug is necessarily more effective, since it is not known exactly which component of the LMWH causes the effect nor its concentration within the molecule. Alternatively one possible explanation of this different behaviour might be that, although these LMWHs share a similar mechanism of action, they must be considered as distinct compounds whose differences can be explained by comparing methods of preparations, molecular structures and electrostatic charge [42], [43]. Considering these differences, it might also be expected that the different biological activity of tinzaparin and enoxaparin, on aPL-mediated inhibition of angiogenesis and on the intracellular mechanisms regulating this process, depend on their unique polysaccharide chain length spectrums and the total amount of negatively charged polyanions.

In conclusion, we previously reported that aPL contribute to defective placentation in APS patients not only by impairing trophoblast cells functions, but also by decreasing HEEC angiogenesis [44], [12]. We also demonstrated that LMWH is able to reduce the aPL antibody binding to trophoblast cells and to restore *in vitro* placental invasiveness and differentiation [8], [12], [15]. We now show that two LMWHs, tinzaparin and enoxaparin, are able to block aPL-inhibition of HEEC angiogenic behaviour and propose several pathways of action. These observations support the beneficial role of LMWHs, not only on the fetal side of the placenta (trophoblast cells), but also on the maternal one (endometrial endothelial cells), hence supporting the presence of multiple mechanisms through which LMWHs may protect APS pregnancy in the early stages.

In clinical practice several strategies have been proposed to improve the outcome of APS pregnancy, including combinations of aspirin, UFH and/or LMWH. However, few well designed trials have been carried out, thus there is no clear evidence about which treatment regimen should be preferred and whether UFH and LMWH have a comparable efficacy. LMWH preparations differ in their methods of production, molecular weight, half-life and anticoagulant activity. Notably, the optimal LMWH has also to be defined. Although LMWHs represent a treatment for prevention of pregnancy complications in APS, little is known about the cellular mechanisms by which heparin exerts its effect on placental tissue. Our investigations provide an important mechanism by which LMWHs are able to prevent the aPL mediated placental damage. Additional *in vitro* and *in vivo* studies are needed to confirm our preliminary results demonstrating the predominant tinzaparin effect in restoring *in vitro* angiogenesis. The outcome of these studies should support large randomized placebo-controlled trials assessing the efficacy of LMWH preparations in the prevention of pregnancy complications associated with APS.
